# Enhanced inflammation and suppressed adaptive immunity in COVID-19 with prolonged RNA shedding

**DOI:** 10.1038/s41421-022-00441-y

**Published:** 2022-07-25

**Authors:** Xiaohua Tang, Rui Sun, Weigang Ge, Tingting Mao, Liujia Qian, Chongquan Huang, Zhouyang Kang, Qi Xiao, Meng Luo, Qiushi Zhang, Sainan Li, Hao Chen, Wei Liu, Bingjie Wang, Shufei Li, Xiaoling Lin, Xueqin Xu, Huanzheng Li, Lianpeng Wu, Jianyi Dai, Huanhuan Gao, Lu Li, Tian Lu, Xiao Liang, Xue Cai, Guan Ruan, Fei Xu, Yan Li, Yi Zhu, Ziqing Kong, Jianping Huang, Tiannan Guo

**Affiliations:** 1 Wenzhou Central Hospital, Dingli Clinical Medical School of Wenzhou Medical University, Wenzhou, Zhejiang China; 2 Department of Genetics and genomic medicine, Zhejiang Provincial people’s hospital (Affiliated People’s Hospital, Hangzhou Medical College), Hangzhou, Zhejiang China; 3 Westlake Intelligent Biomarker Discovery Lab, Westlake Laboratory of Life Sciences and Biomedicine, Hangzhou, Zhejiang China; 4 School of Life Sciences, Westlake University, Hangzhou, Zhejiang China; 5 Key Laboratory of Structural Biology of Zhejiang Province, School of Life Sciences, Westlake University, Hangzhou, Zhejiang China; 6 Center for Infectious Disease Research, Westlake Laboratory of Life Sciences and Biomedicine, Hangzhou, Zhejiang China; 7 Institute of Basic Medical Sciences, Westlake Institute for Advanced Study, Hangzhou, Zhejiang China; 8 Westlake Omics (Hangzhou) Biotechnology Co., Ltd., Hangzhou, Zhejiang China; 9 Zhejiang University School of Medicine, Hangzhou, Zhejiang China; 10 Calibra Lab in DIAN Diagnostics, Key Laboratory of Digital Technology in Medical Diagnostics of Zhejiang Province, Hangzhou, Zhejiang China; 11 Department of Anatomy, College of Basic Medical Sciences, Dalian Medical University, Dalian, Liaoning China; 12 Department of Anatomy and Physiology, College of Basic Medical Sciences, Shanghai Jiao Tong University, Shanghai, China

**Keywords:** Proteomics, Innate immunity

## Abstract

Little is known regarding why a subset of COVID-19 patients exhibited prolonged positivity of SARS-CoV-2 infection. Here, we found that patients with long viral RNA course (LC) exhibited prolonged high-level IgG antibodies and higher regulatory T (Treg) cell counts compared to those with short viral RNA course (SC) in terms of viral load. Longitudinal proteomics and metabolomics analyses of the patient sera uncovered that prolonged viral RNA shedding was associated with inhibition of the liver X receptor/retinoid X receptor (LXR/RXR) pathway, substantial suppression of diverse metabolites, activation of the complement system, suppressed cell migration, and enhanced viral replication. Furthermore, a ten-molecule learning model was established which could potentially predict viral RNA shedding period. In summary, this study uncovered enhanced inflammation and suppressed adaptive immunity in COVID-19 patients with prolonged viral RNA shedding, and proposed a multi-omic classifier for viral RNA shedding prediction.

## Introduction

COVID-19, a disease caused by the severe acute respiratory syndrome coronavirus 2 (SARS-CoV-2), is an ongoing pandemic spreading all over the world. The study on the process of viral RNA shedding is helpful to deepen our knowledge of viral infections and the recovery of human body from a morbid state. Studies have reported that the median of SARS-CoV-2 RNA shedding course is from 10 to 22 days^1–3^, which is usually longer than the duration of symptomatic relief. Remarkably, a case study reported that viral RNA shedding could be over 151 days^4^. Another individual with COVID-19 was reported to be infectious for over 70 days, and its viral RNA shedding course lasted for over 105 days after the initial diagnosis^5^. Prolonged RNA shedding mostly occurs in carriers with increased contagion risk, who are usually elderly, male, or with comorbidities such as hypertension^6,7^. The immunosuppression and some comorbidities have been reported to increase the risk of prolonged viral RNA shedding in other infectious diseases^8,9^. A deeper understanding of the viral shedding mechanisms is, therefore, crucial to help develop better strategies to control the spread of SARS-CoV-2.

In-depth proteomics and metabolomics technologies provide highly detailed and comprehensive molecular expression data shedding light on the underlying physio/pathological processes. Multiple correlational studies, based on proteomics or metabolomics, have characterized circulating molecular changes in patients with severe and non-severe COVID-19^10–14^. Little is known, however, on the molecular modulation in patients with prolonged RNA shedding.

Here we report a systematic and longitudinal clinical and molecular landscape of the COVID-19 patients with long and short RNA shedding courses (LC and SC groups), including 1252 proteins and 945 metabolites across 461 serum samples. We further built a model to predict the persistence of SARS-CoV-2 RNA shedding. In summary, this study not only presents a rich data resource for studying the COVID-19 host responses but also proposes potential diagnostic and therapeutic strategies for COVID-19 patients with prolonged viral RNA positivity.

## Results

### Prolonged RNA shedding in COVID-19

**Fig. 1:**
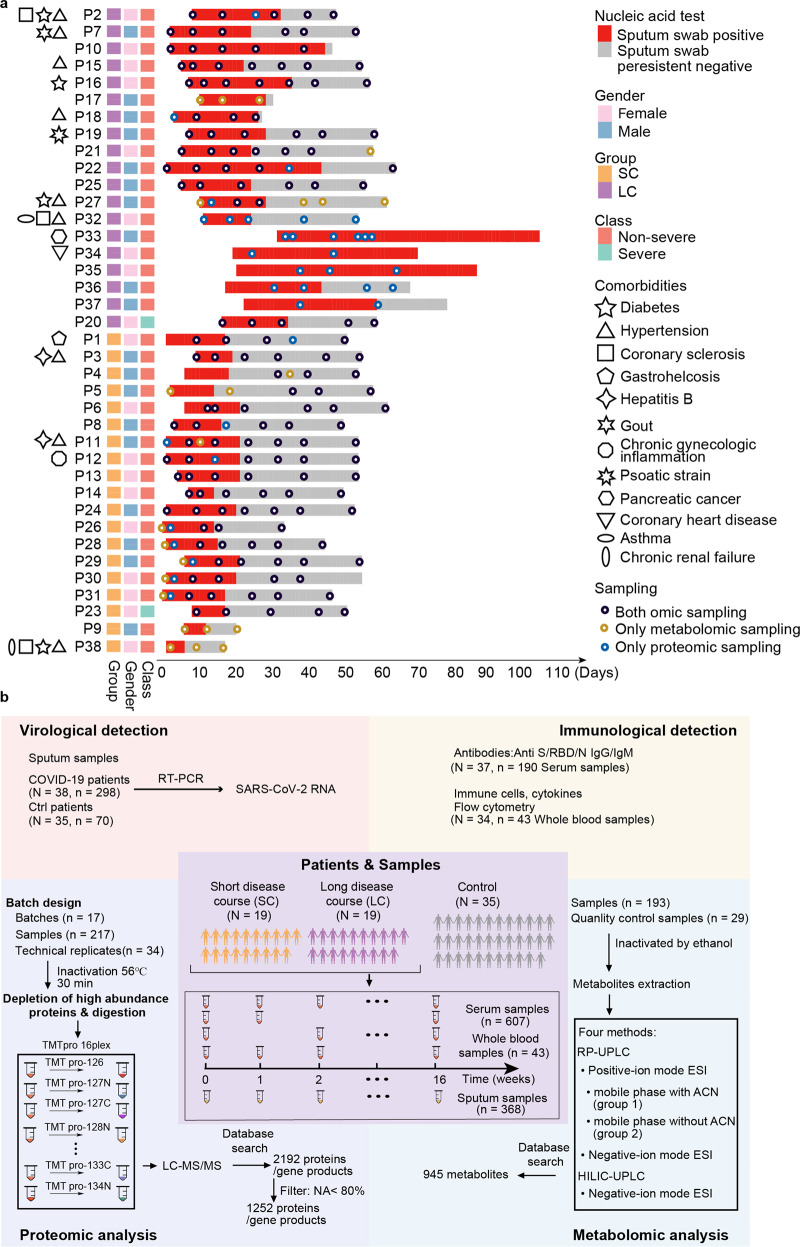
Patients, samples, and study workflow. **a** An overview timeline of our study cohort. The *y*-axis shows the patient ID, and the *x*-axis displays the length of RNA shedding measured from the onset. The 38 patients included 19 with short and 19 with long shedding courses (SC and LC, respectively). Other important information, including the virus nucleic acid test results (sputum or throat swab positive/negative result), gender, severity, comorbidities, etc., are shown in the right panel of the figure. The sputum swab was marked as positive from the first to the last continuously positive test during our observation; a persistent negative swab was negative until the end of our observation. The black dots indicate the sampling time for both omics data, while the blue or orange dots represent only the proteomics or metabolomics data, respectively. More details are provided in Supplementary Table S1. **b** Multi-omics overview involving virological detection based on RT-PCR, an immunological assay based on ELISA and flow cytometry, proteomics and metabolomics analyses for 38 COVID-19 patients and 35 control patients. A total of 298 sputum swab samples (from the 38 COVID-19 patients) and 70 sputum swab samples (from the 35 control patients) were used for the SARS-CoV-2 RNA assay across 16 weeks. Immunological measurements were comprised of 190 serum samples for antibodies-mediated detection over nine weeks and 43 whole blood samples for immune cell counting over three weeks. 217 serum samples and 251 peptide samples, including 34 technical replicates, were analyzed by TMT 16-plex-based quantitative proteomics. 193 serum samples and additional 29 quality control samples for metabolomics analysis were acquired with four different methods including three types of RP-UPLC and one of HILIC-UPLC. A total of 945 metabolites were identified.

In this study, 38 COVID-19 patients were enrolled, including 36 mild cases and 2 severe ones (Fig. 1, Table 1; Supplementary Table S1). To identify the factors responsible for the prolonged viral RNA shedding, we stratified these patients to two groups based on their viral RNA shedding time. In the literature, when Yan et al. analyzed the clinical factors associated with the viral RNA shedding, they used the median, i.e., 23 days, as the cutoff to stratify their patients into the long and short groups^6^, while Xu et al. used the median shedding time, i.e., 17 days, to classify the different patients^7^. Following these conventions, we used the median viral RNA shedding time, i.e., 22.5 days, as the threshold to split the patients into the LC and SC groups. Thus, these patients were split into two groups based on the viral RNA shedding duration (Fig. 1). The SC group contained 19 patients with RNA shedding courses shorter than 22.5 days, while the remaining 19 patients were placed into the LC group. This cutoff was very close to a previous paper^6^. Of the two severe cases, one belonged to the SC group, while the other fell into the LC group (Fig. 1). No significant difference was found in age, gender, comorbidities, drug treatment, or routine blood tests between the LC and SC groups (Supplementary Fig. S1). Notably, five patients exhibited surprisingly prolonged RNA shedding, and the longest duration that we have observed was 110 days (P33, a 55-year-old male) as of 20 May 2020. We therefore reallocated these patients, whose viral RNA positivity persisted longer than 44 days (corresponding to the 3rd quartile of the shedding duration in the LC group) to the very long RNA shedding (LLC) subgroup. Data from these five patients were then used to study the molecular rewiring associated with very long RNA shedding.

**Table 1:** Clinical characteristics of the studied cohort.

Baseline characteristic	Non-COVID-19	COVID-19 SC	COVID-19 LC
	(*N* = 35)	(*N* = 19)	(*N* = 19)
Gender—no.^a^(%)			
Male	18 (51.4)	9 (47.4)	10 (52.6)
Female	17 (48.6)	10 (52.6)	9 (47.4)
Age—yr^b^			
Mean ± SD	43.1 ± 18.3	42.9 ± 12.5	51.5 ± 11.5
Median (IQR)	37.0	44	51
	(28.5–56.0)	(33.0–50.0)	(44.5–56.0)
Range	18–80	20–67	33–84
Symptoms—no. (%)			
Fever	29 (82.9)	10 (57.8)	13 (68.4)
Cough	2 (5.7)	13 (73.7)	17 (89.5)
Diarrhea	1 (2.9)	11 (63.2)	8 (42.1)
Fatigue	1 (2.9)	8 (47.4)	8 (42.1)
Comorbidities—no. (%)			
Hypertension		3 (15.8)	6 (31.6)
Diabetes		1 (5.3)	3 (15.8)
Hepatitis B		2 (10.5)	0 (0.0)
Coronary sclerosis		1 (5.3)	3 (15.8)
Gastrohelcosis		1 (5.3)	0 (0.0)
Psoatic strain		0 (0.0)	1 (5.3)
Chronic gynecologic inflammation		1 (5.3)	0 (0.0)
Gout		0 (0.0)	1 (5.3)
Asthma		0 (0.0)	1 (5.3)
Chronic renal failure		1 (5.3)	0 (0.0)
Treatment—no. (%)			
Lopinavir and ritonavir		19 (100.0)	19 (100.0)
Atomized interferon		19 (100.0)	19 (100.0)
Arbidol		7 (36.8)	12 (63.2)
Lianhuaqingwen (Chinese traditional medicine)		16 (84.2)	17 (89.5)
Ribavirin		0 (0.0)	3 (15.8)
Hydroxychloroquine		1 (5.3)	3 (15.8)

^a^no.: number.

^b^yr.: year.

### Delayed and sustained increase of IgG, elevated cytokines and Treg cells associated with prolonged RNA shedding

We first compared the SARS-CoV-2 viral load in the sputum of the patients at admission between the SC and LC groups, and observed no difference (*P* = 0.28) (Supplementary Fig. S2a). The finding suggests that the discrepancy of RNA shedding might be due to the host responses rather than the viral load.

**Fig. 2:**
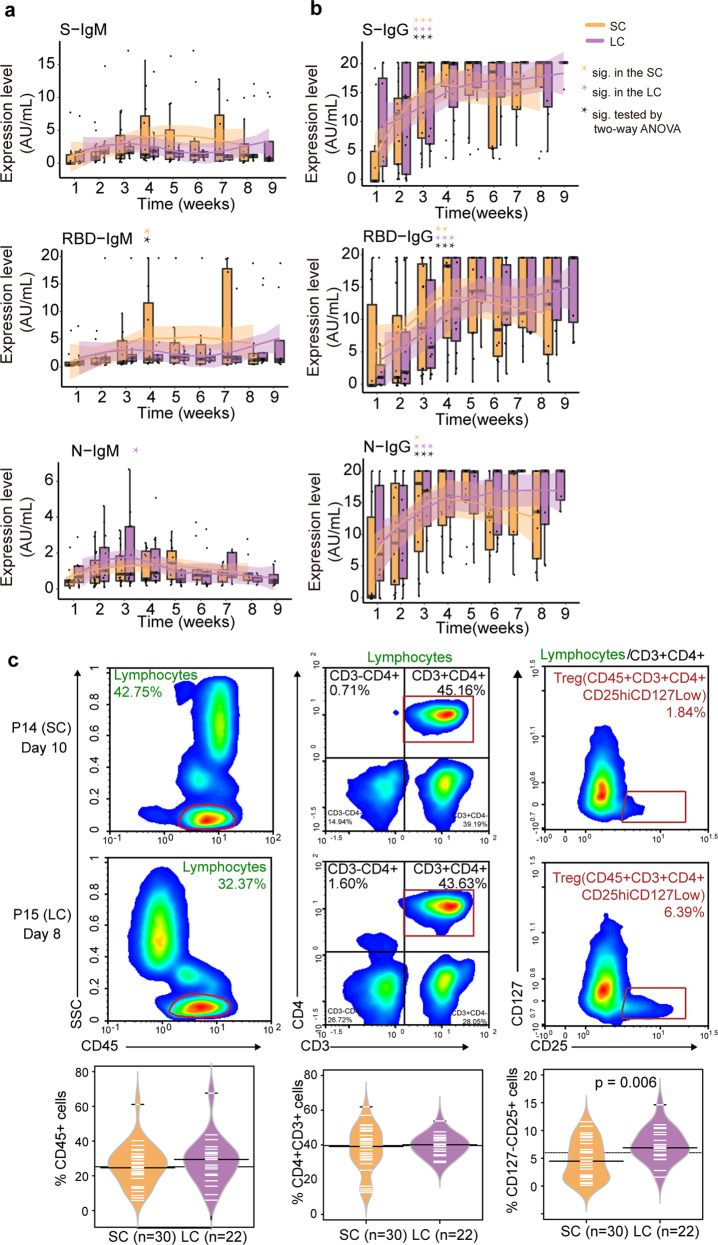
Clinical characteristics of the two groups. **a**, **b** Comparative quantification of six antibody categories between the LC and SC groups. Number of samples collected for each week: 10 SC and 8 LC in week 1, 16 SC and 13 LC in week 2, 16 SC and 13 LC in week 3, 11 SC and 15 LC in week 4, 13 SC and 9 LC in week 5, 10 SC and 11 LC in week 6, 8 SC and 5 LC in week 7, 9 SC and 9 LC in week 8, 8 LC in week 9; total samples: 93 from 17 SC patients, and 91 from 19 LC patients. The profiles of the serum anti-S, anti-RBD, and anti-N IgM (**a**) and IgG (**b**) between the two groups along time. Orange asterisks mean significant variance over 8–9 time points in the SC group (one-way ANOVA). Purple asterisk means significant variance over 8–9 time points in the LC group (one-way ANOVA). Black asterisks show the interactional difference between time points and LC/SC group (two-way ANOVA). *P < 0.05; **P < 0.01; ***P < 0.001. **c** Flow cytometry analysis of immune cells between the SC and LC patients. 22 samples from 17 LC patients and 30 samples from 17 SC patients were analyzed. Flow cytometry analysis of lymphocytes, CD4^+^ cells and CD127^–^CD25^+^ Treg cells of two representative patients (*P*-value from Welch’s *t*-test). The bean plots show the comparison between the two groups.

We then analyzed the plasma cytokines with previously reported clinical importance, namely TNF-α, IFN-γ, IL-6, IL-2, and IL-4, using flow cytometry. Higher expression of cytokines was detected in most LC patients (Supplementary Fig. S2b). Next, we measured different types of IgM (Fig. 2a) and IgG (Fig. 2b) targeting the three viral proteins or domains, namely the spike (S) protein, the receptor-binding domain (RBD) of the S protein, and the nucleocapsid (N). Notably, we found that the IgG antibody expression was significantly different between the SC and LC groups across the nine weeks (Fig. 2a, b). Our data showed that the lgG level increased in both groups during the first five weeks, and decreased only in the SC patients afterwards (Fig. 2b).

We also found that the number of CD127^Low^CD25^High^ Treg cells was significantly higher in the LC patients than that in the SC patients (*P* = 0.006), while the CD45^+^ lymphocytes (*P* = 0.306) and the CD3^+^CD4^+^ T cells (*P* = 0.871) showed no significant difference (Fig. 2c).

### Temporal proteomic and metabolomic profiling of serum

To further characterize the underlying molecular mechanisms responsible for prolonged RNA shedding, we performed in-depth proteomic and metabolomic profiling of 217 serum samples derived from the 38 COVID-19 patients and 35 non-COVID-19 controls (Ctrl) over nine weeks since the disease onset.

A total of 2192 proteins were quantified by tandem mass tag (TMT)-based proteomics (Supplementary Table S2a). The batch effect was negligible (Supplementary Fig. S3a–d). The median coefficient of variance (CV) among technical replicates was 15% (Supplementary Fig. S3g). After excluding proteins with over 80% missing values, 1252 proteins were subjected to downstream data analysis (Supplementary Table S2a).

Metabolomic analysis characterized 945 metabolites in 193 serum samples from the same patient cohort using both hydrophilic and hydrophobic molecules analyzed by both positive and negative ionization modes (Supplementary Table S3a). Batch effect was negligible (Supplementary Fig. S3e, f), and the median CVs of four methods among 29 technical replicates were all below 12% (Supplementary Fig. S3h), indicating high quality of the data.

### Delayed immune response in the LC group

**Fig. 3:**
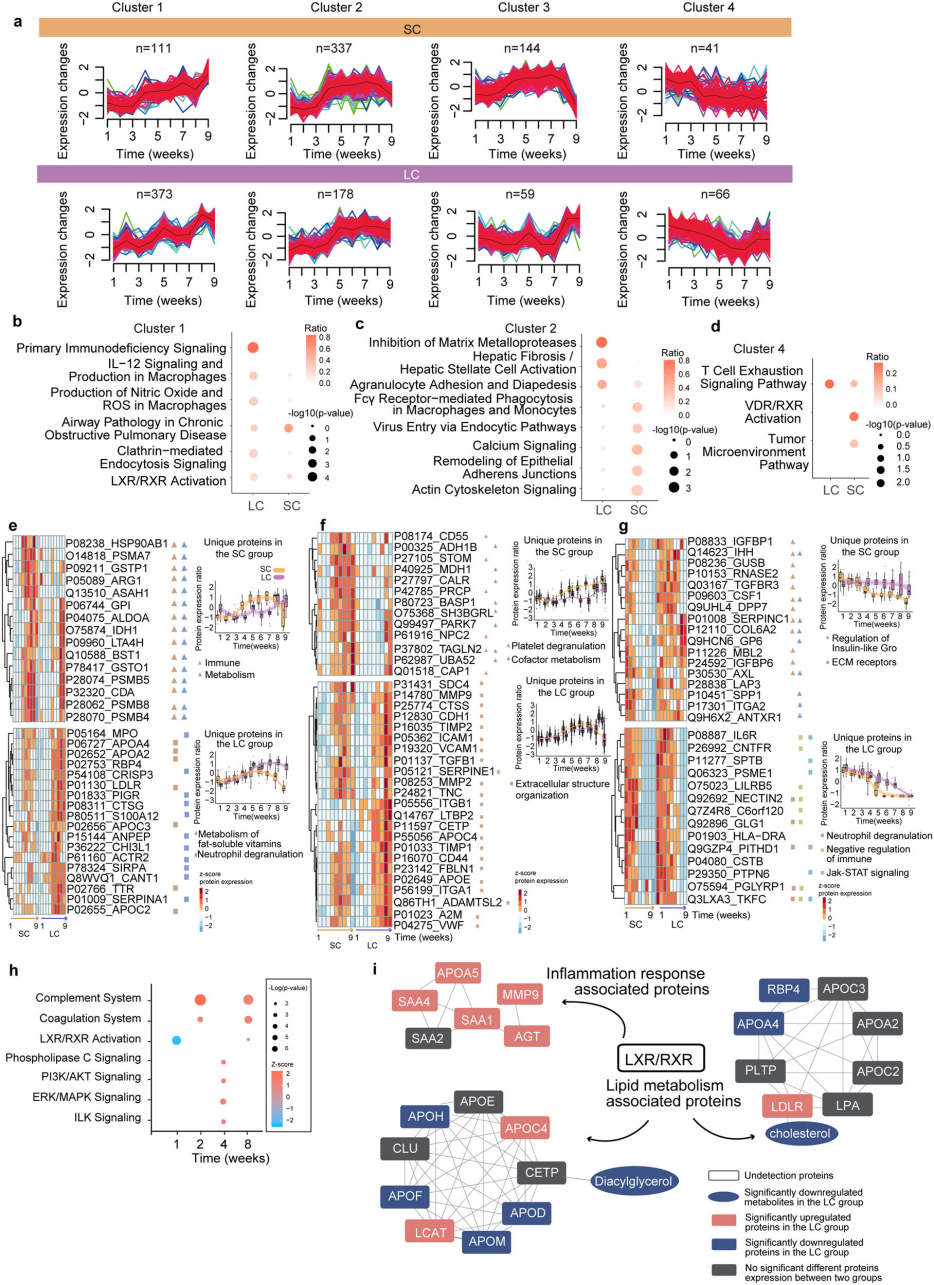
Dynamic proteomics profiling. **a** The four clusters of proteins with different expression dynamics for the SC and LC groups computed with Mfuzz analysis (one-way ANOVA, B-H adjusted *P*-value < 0.05, more details in Supplementary Table S2b). **b**–**d** Pathways enrichment by ingenuity pathway analysis (IPA) for the proteins in Clusters 1, 2, and 4 of the SC and LC groups. **e**–**g** Heatmaps showing the unique proteins of the SC and LC groups in Clusters 1, 2, and 4 (more details in Supplementary Table S2b), as well as the corresponding top two pathways annotated by IPA (*P*-value < 0.05). Proteins highlighted in red are discussed in the main text. **h** Pathways enriched by IPA (*P*-value < 0.05) across the nine weeks (no pathways were enriched for the 3rd, 5th, or 9th weeks) using the differentially expressed proteins between the LC and SC groups (Supplementary Table S2d). The *z*-score represents the activation state of the pathways: *z*-score > 0 means the pathway is active, while *z*-score < 0 indicates that the pathway is inhibited. **i** The relationship between LXR/RXR and their downstream proteins which uniquely belong to the LC (lipid metabolism-associated proteins) and SC (inflammation response-associated proteins) groups in Cluster 1 (**a**). Blue: downregulated proteins/metabolites in the LC group at the 1st week; red: upregulated proteins/metabolites in the LC group at the 1st week; gray: no significantly different molecules were found between the two groups at the 1st week. The interactions retrieved from STRING are visualized with Cytoscape.

To identify the potential molecules responsible for longer viral RNA shedding courses, we compared the temporal proteomes of the SC and LC patients. Four dynamic clusters (Supplementary Table S2b) and their enriched pathways were portrayed in the SC and LC groups, respectively (Fig. 3a–d). Interestingly, three clusters in LC patients showed similar dynamics with three respective clusters in the SC group. Next, we focused on these three pairs of clusters. Proteins from Cluster 1 displayed a consistently ascending pattern till the 9th week; for this cluster, primary immunodeficiency signaling was the most significantly and exclusively enriched pathway in the LC group (Fig. 3b; Supplementary Table S5a). This finding is supported by a previous study reporting immunodeficiency in a COVID-19 case with long viral RNA shedding^5^. In Cluster 2, the dynamic proteome tended to keep ascending till the 5th week and the pattern was relatively delayed in the LC group. Disease-associated pathways were significantly enriched in the LC group, whereas the SC group was mainly characterized by tissue remodeling and cytoskeleton-associated remodeling pathways (Fig. 3c; Supplementary Table S5a). The results suggest that the tissue damage was more severe in the LC group, while remodeling occurred in the SC group. Regarding Cluster 4, the turning point for the LC group appeared ~2 weeks later than that for the SC group. T cell exhaustion was more significantly enriched in the LC group (Fig. 3d; Supplementary Table S5a), indicating that the T cell deficiency is more severe in the LC group.

We next identified the SC/LC group-specific proteins, for each of the three clusters, among those enriched in their top three Reactome pathways (false discovery rate (FDR) *q*-value < 0.05, Fig. 3e; Supplementary Table S5b). In Cluster 1, the proteins that were uniquely clustered in the SC group were all involved in immune response and metabolism, while the fat-soluble vitamin metabolism and neutrophile degranulation pathways were enriched in the LC group (Fig. 3e).

In Cluster 2, proteins associated with platelet degranulation and cofactor metabolism were uniquely enriched in the SC group (Fig. 3f). These proteins were upregulated earlier and persisted longer at a high level in the SC group, indicating a prompt and stable innate response. Proteins associated with extracellular structure organization, on the other hand, were enriched in the LC group (Fig. 3f). They were upregulated with a delay and maintained at a high level until the 9th week, indicating that a delayed tissue remodeling took place in the LC group.

Regarding the consistently descending proteins from Cluster 4, those associated with the insulin-like growth factor (Gro) and extracellular matrix (ECM) receptors were enriched in the SC group (Fig. 3g). Proteins involved in neutrophil degranulation, negative regulation of immunity, and Jak-STAT signaling pathway were significantly enriched in the LC group (Fig. 3g).

Interestingly, the protein dynamics in Cluster 3 was different between the LC and SC groups. In Cluster 3 in the SC patients, 144 proteins enriched mainly in hepatic fibrosis/hepatic stellate cell activation and other five inflammatory pathways (including complement system, IL-15 signaling, acute phase response signaling, LXR/RXR activation pathway, and coagulation system) declined in the weeks 8 and 9, suggesting recovery from this disease. In contrast, 59 proteins in Cluster 3 in the LC patients increased in the LC group. These proteins were uniquely enriched in four pathways, including leukocyte extravasation signaling, calcium signaling, actin cytoskeleton signaling, and axonal guidance signaling (Supplementary Table S5a), suggesting delayed tissue repair.

We further investigated the dynamics of COVID-19-specific molecules by comparing with the control group. These dysregulated molecules showed similar perturbation patterns to those revealed by overall analysis above (Supplementary Table S2c).

Together, our data show that for the innate immunity, the SC group exhibits a prompt and adequate innate response, while the LC group shows a delayed but persistent innate response and tissue remodeling. As for the adaptive immunity, the LC group uniquely shows T cell exhaustion, which might contribute to the suppression of virus clearance.

### LXR/RXR-mediated lipid regulation and innate immunity in the LC group

To further investigate the dysregulated proteins and pathways between the LC and SC groups of patients over nine weeks, we performed a pairwise comparison of the proteomes of both groups for each time point. The dysregulated proteins, identified at each timepoint, were combined to generate a list of 295 significantly dysregulated proteins between the LC and SC groups (Supplementary Table S2d). These 295 proteins could be used to separate the LC and SC samples to various degrees at different time points (Supplementary Fig. S4a). Enriched pathways for these 295 proteins were related to immunity and metabolism (B-H adjusted *P*-value < 0.01) (Fig. 3h).

LXR/RXR activation pathway was found to be significantly inhibited in the LC group in the 1st week (Fig. 3h). In the following weeks, significant activation of the complement and coagulation system was observed in the LC group. Cell proliferation-associated pathways including PI3K/AKT, ERK/MAPK, ILK, and phospholipase C signaling pathways were activated in the 4th week. In the 8th week, complement and coagulation systems were activated again in the LC group (Fig. 3h), providing evidence for ECM remodeling. Inversely, the LXR/RXR pathway was activated in the 8th week. We found downregulation of lipid metabolism-associated molecules, including RBP4, APOA4, APOF, diacylglycerol and cholesterol in the LC group (Fig. 3i), in agreement with the positive regulatory impact of LXR/RXR on lipid metabolism^15^. Furthermore, in our data, acute phase factors including SAA4, SAA1, and AGT were upregulated in the LC group at the 1st week (Fig. 3i), while TNF-α was upregulated in the LC group at the 2nd week (Supplementary Fig. S2b). This observation is supported by findings that LXR/RXR inhibits innate immune response^16^, and that LXR/RXR is inhibited by proinflammatory factors including IL-1β and TNF-α^17^. The innate immune activation induced by LXR/RXR inhibition might have contributed to prolonged viral RNA shedding.

### Prolonged LXR/RXR inhibition contributes to the LLC group

A specific subgroup of patients included in this dataset exhibited unusually long RNA shedding persistence (over 44 days). To examine the molecular characteristics of these patients, we divided the LC group into LLC (longer length of RNA shedding course, over 44 days, which was the 3rd quartile of duration in the LC group, *N* = 5 patients, *n* = 11 samples) and MLC (medium length of RNA shedding course, from 23 to 44 days, *N* = 14 patients, *n* = 56 samples) groups. Serum samples included in this analysis were collected from the 4th week to the 9th week because the LLC group had only one sample collected in the first three weeks due to biosafety issues.

We compared the protein expression in LLC, MLC, and SC groups pairwise (Supplementary Table S2e, f). Interestingly, among 383 dysregulated proteins between the LLC and SC groups, 268 dysregulated proteins were shared with those dysregulated between the LC and SC groups (Supplementary Table S2d), suggesting that the comparison of LC and SC well recapitulated the difference between LLC and SC. Pathway analysis showed that the 268 commonly dysregulated proteins were enriched in 19 pathways (Supplementary Fig. S4b and Table S5c). The remaining 115 proteins were enriched in 18 pathways, with 16 overlapped with the previous 19 pathways, further consolidating the similarity between LC and LLC. Two unique pathways were characteristic for the LLC patients, namely wound healing signaling pathway and inhibition of matrix metalloproteases (Supplementary Fig. S4b and Table S5c), suggesting higher degree of tissue injury and repair in these patients. Of note, the LXR/RXR pathway was again the most significantly inhibited pathway in the LLC group compared with SC or MLC, and the period of its inhibition lasted longer in the LLC group than that in the LC group (Supplementary Table S5c), further consolidating that inhibition of LXR/RXR might have contributed to prolonged viral RNA shedding.

### Dynamic metabolomic profiling reveals downregulation of metabolites in the LC group

**Fig. 4:**
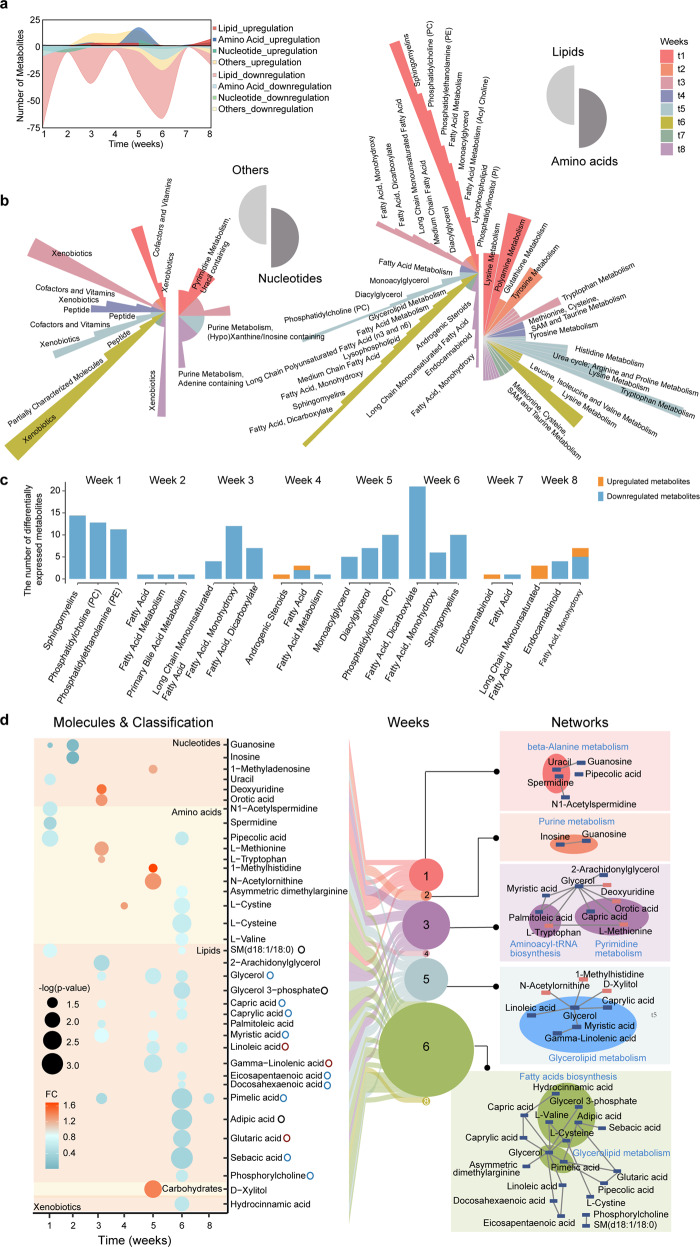
Dynamic metabolomics profiling. **a** Stream graph showing the differentially expressed metabolites, split into four categories (lipid, amino acid, nucleotide, and others), between the SC and LC groups (|Log_2_(fold change (FC))| > 0.25, Welch’s *t*-test *P* < 0.05). A positive number of metabolites represents their upregulation while a negative number represents their downregulation in the LC group. **b** The sub-classifications of the differentially expressed metabolites in four subcategories (as in **a**). The length of the circular sector represents the number of metabolites belonging to a sub-pathway. Sub-pathways containing > 1 metabolite are annotated. **c** The histogram showing that the top three or two dysregulated metabolites between the LC and SC groups in each week. Orange: upregulated metabolites in the LC group; blue: downregulated metabolites in the LC group; |Log_2_(FC)| > 0.25, Welch’s *t*-test *P* < 0.05). **d** Connection between the differentially expressed metabolites, the time-point, and the enriched KEGG pathways. Left: bubble plot showing the differentially expressed metabolites between the LC and SC groups (FC > 1 represents upregulation, whereas FC < 1 represents downregulation, in the LC group); the size of the bubbles represents the degree of significance of the difference between the LC and SC groups. Middle: the sizes of the circles represent the numbers of the differentially expressed metabolites at each week. Right: KEGG pathways annotated with the deeper background-colored circle. Blue or red rectangles represent downregulated or upregulated metabolites in the LC group, respectively. The interaction plot was generated using MetaboAnalyst.

Across the entire disease course, we found that most dysregulated metabolites were downregulated in the LC group (Supplementary Table S3b), including lipids, amino acids, and nucleotides (Fig. 4a, b; Supplementary Fig. S5). Lipids were the most dysregulated metabolites (Fig. 4a, b; Supplementary Fig. S5). The substantial downregulation of lipids has also been observed in severe COVID-19 patients^10,18^. The most significantly downregulated lipids in the LC group are sphingomyelins, phosphatidylcholine (PC), and phosphatidylethanolamine (PE) in the 1st week (Fig. 4c), followed by downregulation of fatty acids and their oxidative products, such as monohydroxy and dicarboxylate fatty acids in the 3rd week (Fig. 4c). PC and sphingomyelins were also the most dysregulated lipids in the 5th and 6th weeks, respectively (Fig. 4c). PC is well-known as an anti-inflammation factor^19^. Its suppression in the LC patients suggests activation of inflammation.

Pathway analysis of the most dysregulated metabolites in the LC group during the first three weeks showed the enrichment of nucleotide metabolism and beta-alanine metabolism (Fig. 4d). Metabolites regulated in the 5th and 6th weeks were mainly fatty acids (Fig. 4d). Among the downregulated metabolites were anti-inflammation molecules, such as eicosapentaenoic acid (EPA), docosahexaenoic acid (DHA), capric acid, and caprylic acid^20,21^ (Fig. 4d). This indicates persisted inflammation in the LC patients.

### Activated complement system, suppressed cell migration, and enhanced viral replication plausibly contribute to prolonged RNA shedding

**Fig. 5:**
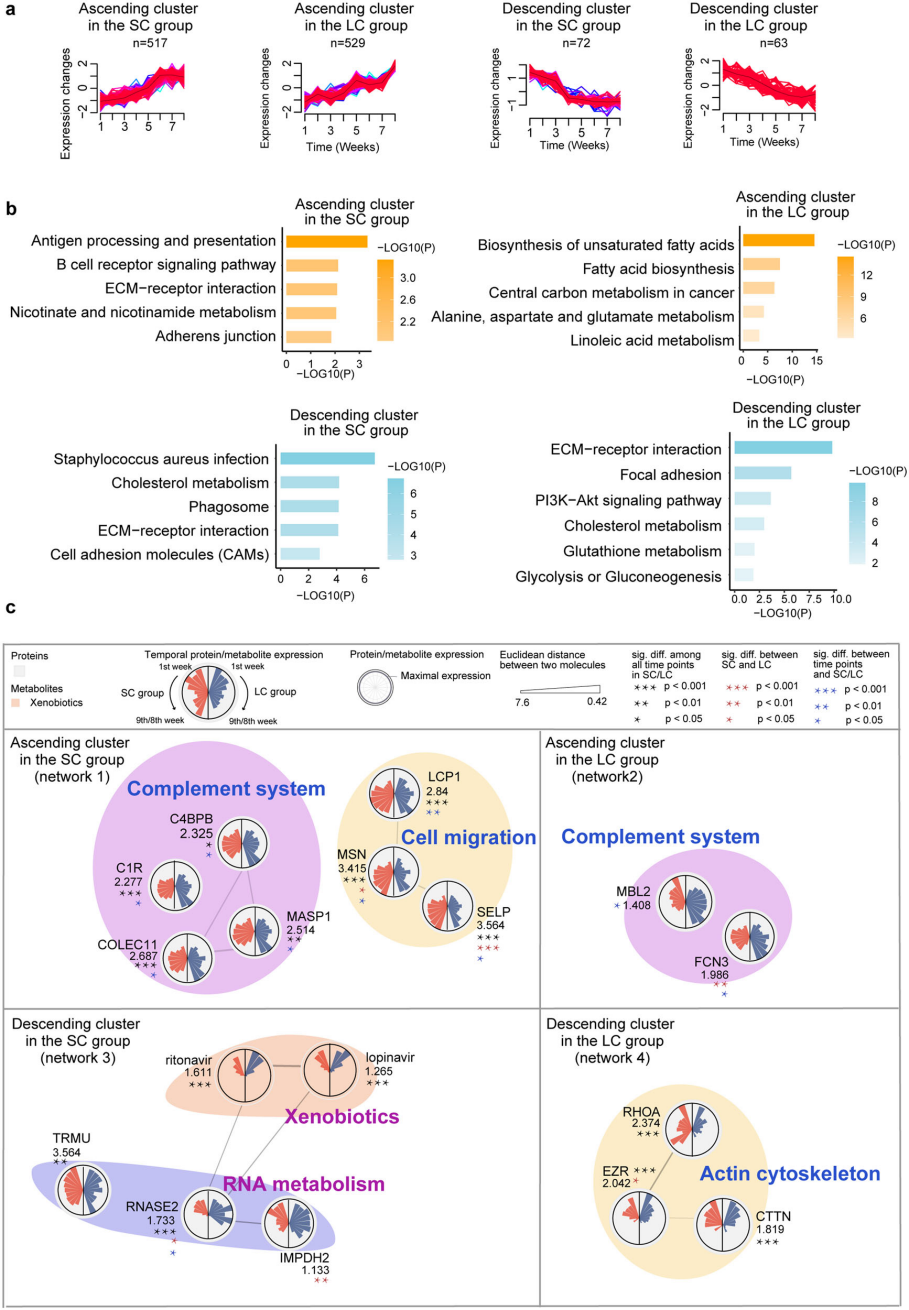
Integrative analysis of proteome and metabolome. **a** Four clusters of proteins with different protein and metabolite expression dynamics for the SC and LC groups computed using Mfuzz (one-way ANOVA, B-H adjusted *P*-value < 0.05). **b** Pathways enriched using MetaboAnalyst (*P*-value < 0.05). **c** The annotated proteins are shown in the four networks. For each circle, the right/left half panel shows the expression time-series in the LC/SC group (two-way ANOVA; *P < 0.05; **P < 0.01; ***P < 0.001). Black asterisks indicate a significant variance over 8–9 time points in the SC or LC groups; the red asterisks represent the difference between the LC and SC groups; the blue asterisks represent the interaction difference between time points and the LC and SC groups. The outermost ring represents the maximum abundance of the proteins/metabolites, between the SC and LC groups, across nine time points for proteins and eight time points for metabolites. The different backgrounds represent the classification of the molecules.

We next identified persistently ascending and descending molecules using Mfuzz in the SC and LC patient groups, respectively. This resulted in four clusters (Fig. 5a; Supplementary Table S4). We then applied ingenuity pathway analysis (IPA) to the molecules, and found that the most significant pathways enriched from ascending molecules in the SC group were antigen processing and presentation, and cell adhesion (Fig. 5b; Supplementary Table S5d). In contrast, ascending molecules in the LC group were enriched for pathways including biosynthesis of unsaturated fatty acids (Fig. 5b). *Staphylococcus aureus* infection ranked first in the descending molecules in the SC group, while ECM–receptor interaction was the most enriched pathway in the descending molecules in the LC group (Fig. 5b). We further built a *k*-nearest neighbors (KNN) network to investigate the molecules involved in the pathways, and identified a few functional groups (Supplementary Fig. S6). Remarkably, complement system proteins, including collectin-11 (COLEC11), MBL-associated serine protease 1 (MASP1), Mannose-binding lectin 2 (MBL2), and Ficolin-3 (FCN3), were persistently highly expressed in the LC group across the entire disease course (Fig. 5c, network 1 and network 2). The activation of complement system may induce severe inflammatory injury of COVID-19 patients as an innate immune response^22^. The data suggest that the prolonged innate immunity accompanied with more severe inflammatory injury might contribute to prolonged disease course, in agreement with prolonged innate immunity response induced by LXR/RXR suppression (Fig. 3).

Recruitment of immune cells is usually promoted by innate immunity. However, we found lower expression of proteins participating in cell migration, including selectin P (SELP), moesin (MSN), and lymphocyte cytosolic protein 1 (LCP1) (network 1 in Fig. 5c). In particular, the prolonged lower expression of MSN in the first seven weeks of the LC disease course suggests a deficiency of lymphocyte egression to kill the pathogen^23^. Ezrin (EZR) exhibits opposite functions in lymphocytes to MSN in the ezrin–radixin–moesin (ERM) complex^24^, and indeed, our data showed higher expression of EZR in the LC group (network 4 in Fig. 5c). These observations together indicate a deficiency in leukocyte migration in the LC patients.

Molecules associated with xenobiotics and RNA metabolism were elevated in the LC group (Fig. 5c). Upregulation of proteins participating in viral RNA metabolism, including a non-secretory ribonuclease (RNASE2)^25^ and Inosine-5’-monophosphate dehydrogenase 2 (IMPDH2)^26^, suggests that viral replication might persist longer in the LC group, and these proteins might be potential therapeutic targets.

Altogether, our KNN-based network analysis uncovered several kinds of biologically important proteins and pathways. These factors, associated with the activation of innate immune response, deficiency in leukocyte migration and longer viral replication, collectively contributed to prolonged RNA shedding in the LC group of patients.

### Predictive model for prolonged viral RNA shedding period

**Fig. 6:**
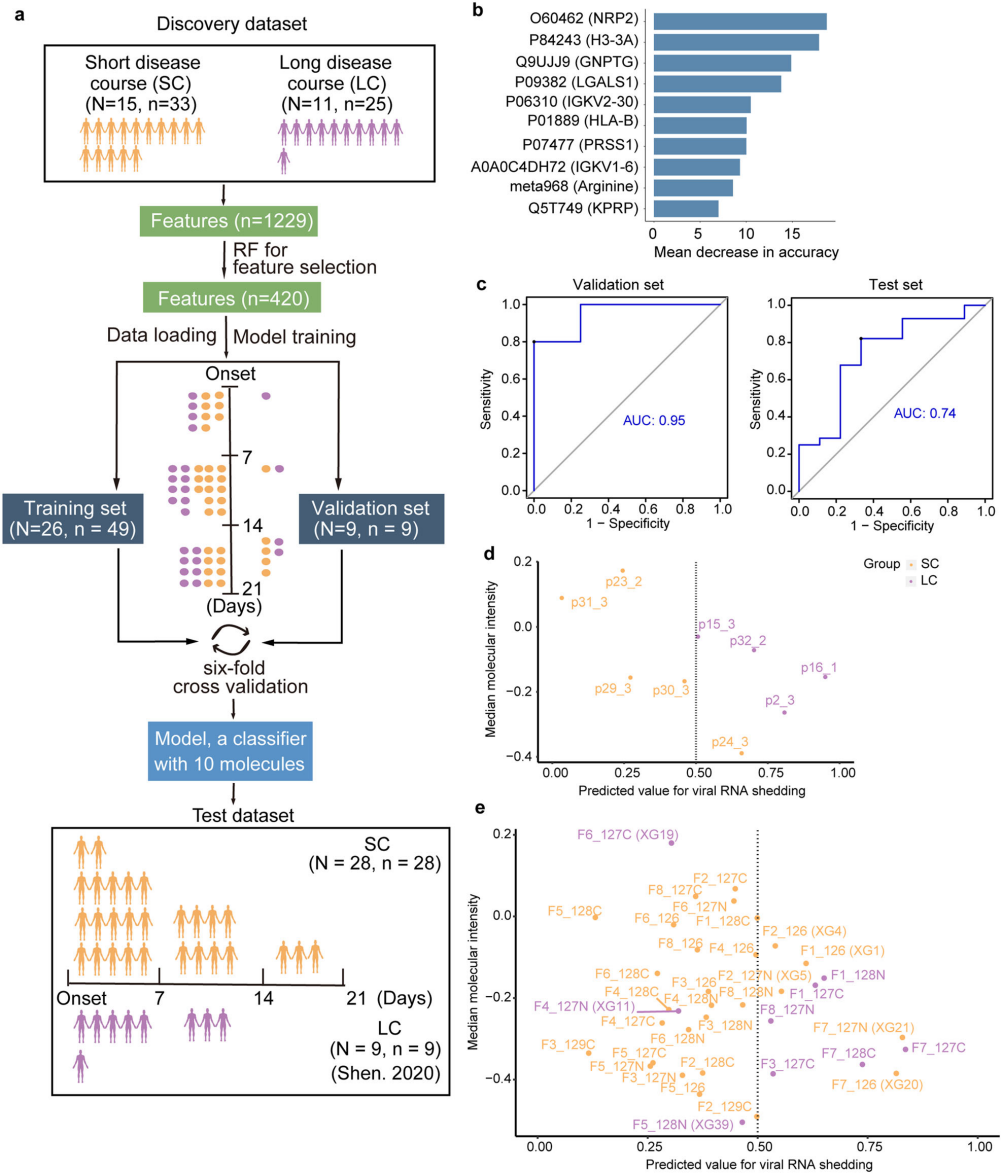
Machine learning model for disease course prediction. **a** Workflow for machine learning; RF random forest. **b** The top ten features selected by the machine learning model. **c** The ROC of validation dataset (left panel) and independent test dataset (right panel). **d**, **e** Performance of the model in the validation (**d**) and the independent test datasets (**e**). Orange represents the SC group and purple represents the LC group.

To predict prolonged viral RNA shedding in COVID-19 patients during the early phase, we developed a machine learning model based on the serum proteomic and metabolomic data collected during the first three weeks (see the Materials and methods section). We included 58 samples from 26 patients with both proteomic and metabolomic data as a discovery dataset. These samples were randomly divided into two groups: a 49-sample training dataset and a 9-sample validation dataset. We also included an independent dataset comprising 37 samples from 37 patients with both proteomic and metabolomic data in the first three weeks (i.e., the Shen dataset^10^) (Fig. 6a).

Based on their expression robustness and the importance prioritized by random forest analysis (more details in the Materials and methods section), we selected nine proteins (NRP2, H3-3A, GNPTG, LGALS1, IGKV2-30, HLA-B, PRSS1, IGKV1-6, KPRP) and one metabolite (arginine) to construct a 10-molecule model (Fig. 6b). Immunoglobulin kappa variable 2–30 (IGKV2–30), immunoglobulin heavy variable 1–6 (IGHV1–6), and HLA class I histocompatibility antigen, B alpha chain (HLA-B) are all associated with antibody secretion and humoral immunity. Notably, two proteins, neuropilin-2 (NRP2) and galectin-1 (LGALS1), have been reported to promote the entry of SARS-CoV-2 virus^27,28^. Arginine is an essential amino acid, promoting T cell proliferation^29^. Several studies showed that arginine is downregulated in the serum of COVID-19 patients^30^.

The area under curve (AUC) values for the training dataset and the validation dataset were 1 and 0.95, respectively (Fig. 6c, d). This model led to only one incorrect prediction in the validation dataset. The SC patient P24 was classified as an LC case. This is probably because this 35-year-old male patient had been treated with an immunomodulatory drug hydroxychloroquine.

In the independent test set, the model correctly classified 29 out of 37 patients with an overall accuracy of 80% (AUC = 0.74, Fig. 6c, e). The incorrect prediction of the rest 8 cases may be attributed to their complex clinical history. XG39, an LC patient, developed severe symptom on the day of sampling, which might influence the performance of the model. The immunosuppression status of the XG20 patient with diabetes and the XG1 case with splenectomy may have misled the model.

The other five cases, namely XG4, XG5, XG21, XG19, and XG11, exhibited viral RNA shedding periods of 16, 19, 20, 23, 27 days, respectively. The RNA shedding periods were close to the binary classification threshold of 23 days. The incorrect prediction of these cases indicates the complexity of the viral RNA shedding prediction and necessitates future verification of this model in larger sample sets. Altogether, our data suggest that this multi-omic classifier could potentially predict the SARS-CoV-2 RNA shedding.

## Discussion

To understand the molecular mechanisms underlying prolonged viral RNA shedding in COVID-19 patients, we profiled a deep and time-resolved landscape of their plasma proteome and metabolome. Our data showed that these patients exhibited prolonged inflammation and suppressed adaptive immunity. Besides, we found that a 10-molecule model could potentially predict prolonged viral RNA shedding, including NRP2, H3-3A, GNPTG, LGALS1, IGKV2-30, HLA-B, PRSS1, IGKV1-6, KPRP, and arginine.

Our data showed that the LC patients were characterized by prolonged inflammation. First, we detected upregulation of multiple proinflammation cytokines in LC patients, such as TNF-α and IL-6 by antibody-based assay (Supplementary Fig. S2b), and macrophage colony-stimulating factor 1 (CSF1) by MS-based proteomics (Fig. 3g). These cytokines participate in multiple immune responses, including macrophage activation, monocyte recruitment, and antigen response^31^. Moreover, our proteomics data also showed early inhibition of LXR/RXR and activation of complement system in the LC group, which may have contributed to prolonged inflammation. Multiple complement system proteins, including COLEC11, MASP1, MBL2, and FCN3, were elevated in the LC group across the entire disease course (Fig. 5c). In addition, MS-based metabolomic analysis showed downregulation of a large number of anti-inflammation lipids, as well as multiple amino acids (Fig. 4d). Spermidine, a kind of polyamine, has been reported to inhibit synthesis of proinflammatory cytokines^32^ through blocking NF-κb, PI3K/AKT and MAPK pathways^33^. Together, these data suggest that the COVID-19 patients with prolonged viral RNA shedding exhibited characteristically enhanced inflammation.

Our data also showed suppressed adaptive immunity in these patients. Flow cytometric analysis uncovered increased Treg cells in the LC group (Fig. 2c). Treg cells have been implicated with the impairment of the cytotoxic T cell function in defense of viral infection^34^. Thus, the higher level of Treg cells observed in the LC group may contribute to the T cell exhaustion, leading to a suppression of defense against the virus.

Virus infection initiates innate immune response, including release of acute phase proteins and inflammatory cytokines^35^, which stimulates adaptive immunity to eliminate pathogens. Surge of adaptive immune cells tempers the initial innate responses^36^. Thus, the limited adaptive immunity might be insufficient to clean the virus and to suppress prolonged inflammation, leading to prolonged viral RNA shedding. For SARS-CoV-2 and MERS-CoV, prolonged viral RNA shedding has been found in immunocompromised patients^37,38^. For COVID-19, an immunocompromised patient has been reported with over 100 days of viral RNA shedding^5^; however, the underlying molecular mechanisms remain elusive. Here our data showed an increase of Treg cells and prolonged inflammation in COVID-19 patients with prolonged viral RNA shedding.

The 10-molecule model could potentially predict prolonged viral RNA shedding. The nine proteins and arginine participate in multiple immune responses and metabolism processes, suggesting perturbed immunity and metabolism in the COVID-19 patients with viral RNA shedding. To consolidate the MS-based protein identification for IGKV2-30 (Supplementary Fig. S7a) and IGHV1-6 (Supplementary Fig. S7b), we manually inspected the MS/MS spectra of their unique peptides. The data confirmed unambiguous identification of these proteins (Supplementary Fig. S7). Nevertheless, clinical translation of these biomarkers awaits further investigations. This study thus provides a rich data resource to study the longitudinal host response of COVID-19, and it also suggests potential diagnostic and therapeutic strategies for COVID-19 patients with prolonged viral RNA positivity.

Several studies of COVID-19 blood samples have identified multiple regulated proteins and metabolites in severe cases compared with non-severe cases^10–14^. However, no study has been reported to investigate the prolonged viral RNA shedding, neither has any study presented any means to predict the prolonged viral RNA shedding. The innate immune response is enhanced in the severe patients, such as the activation of the acute phase proteins and complement system, and massive decrease of metabolites^10^. This study shows that these pathways are also dysregulated in the LC patients. Remarkably, the LC patients also exhibited more enhanced inflammation, characterized by inhibition of LXR/RXR during the first week since disease onset, and activation of complement and coagulation systems during the 2nd to 8th weeks. The unique characteristic in these LC patients is elevated Treg cells which suggests suppressed adaptive immunity.

This study is limited by the relatively small patient number, in particular the severe cases. However, for each patient we have collected longitudinal samples for dynamic monitoring. Here we procured only five LLC patients with the RNA shedding period of over 44 days; and due to biosafety issues, we did not obtain their samples in the first three weeks, thus unfortunately we could not investigate the predictive power of the machine learning model for the LLC pateints. Nevertheless, our data revealed molecular changes in these patients which might be of value for further investigations of prolonged RNA shedding. Rigorous statistics have been employed to identify significantly disturbed molecular expression and pathway activities. More independent validation cohorts are needed to validate the current RNA shedding prediction model. The diagnostic and therapeutic potential of the findings awaits further investigation. The COVID-19 pandemic is rapidly evolving. By the publication of this paper, the dominant strain of SARS-CoV-2 is Omicron and its variants with evolved pathogenicity. The biological insights and predictive model established here may not be directly applicable to the changing viruses, although our recent proteomic study of the Omicron has uncovered some similarities between this new strain and the original strain. However, the AI-empowered proteomic methodology established here could be directly applied to the current SARS-CoV-2 infections and other infectious diseases. Should more COVID-19 specimens have been properly stored, this study will be able to contribute more to the fight against the ongoing pandemic.

## Materials and methods

### Patients and sera samples

We procured 38 COVID-19 patients and 35 non-COVID-19 patients in January–March 2020 (Fig. 1). Besides, 298 sputum swab samples of 38 COVID-19 patients for 16 weeks and 70 sputum swab samples of non-COVID-19 patients were collected for virological analysis. Moreover, 190 serum samples were used for immunological detection by SARS-CoV-2-specific antibodies, as well as 43 whole blood samples for immune cell counting over 3 weeks. Furthermore, 217 and 193 serum samples were, respectively, collected for proteomic and metabolomic analyses over a timespan of 9 and 8 weeks.

We procured 73 patients in this study, including 38 COVID-19 patients whose sputum swabs were tested positive for SARS-CoV-2 according to the manufacturer’s instructions (Shanghai BioGerm Medical Technology Co., LTD., Shanghai, China). According to the Chinese Government Diagnosis and Treatment Guideline (Trial 4th version), these 38 COVID-19 patients include 36 general cases and two severe cases. We have also procured 35 non-COVID-19 patients showing similar flu-like clinical symptoms to COVID-19 patients who are negative for SARS-CoV-2 as indicated by nucleic acid testing. More detailed information of these patients is provided in Fig. 1a and Supplementary Table S1.

Totally 217 serum samples from these patients were collected longitudinally for proteomics analysis (Fig. 1b; Supplementary Table S1). Sampling was performed in the early morning before diet using serum separation tubes (BD, USA). The blood was clotted for ~30 min at room temperature, and then centrifuged at 1000× *g* for 10 min for serum sample collection. This study has been registered in the Chinese Clinical Trial Registry with an ID of ChiCTR2000031699. The study methodologies conformed to the standards set by the Declaration of Helsinki. The experiments were undertaken with the understanding and written consent of each subject. This study has been approved by the Ethical/Institutional Review Board of Wenzhou Central Hospital and Westlake University.

### Proteomic analysis

Serum samples were prepared as previously described^10^. Briefly, samples were first inactivated and sterilized at 56 °C for 30 min. For proteomics study, 14 high abundant serum proteins were depleted from 4 μL serum samples by diluting into 500 μL PBS using a human affinity depletion kit (Thermo Fisher Scientific™, San Jose, USA), and then concentrated into 50 μL through a 3 K MWCO filtering unit (Thermo Fisher Scientific™, San Jose, USA). The concentrated samples were mixed with 500 μL 8 M urea (Sigma) and concentrated into 50 μL. The samples were then reduced and alkylated with 10 mM tris (2-carboxyethyl) phosphine (TCEP, Sigma) and 40 mM iodoacetamide (IAA), respectively. Proteins were subjected to a two-step tryptic digestion (enzyme to protein ratio: 1:20; Hualishi Tech. Ltd., Beijing, China). The digestion was then stopped by acidification to pH 2–3 by 1% trifluoroacetic (TFA) (Thermo Fisher), and peptides were subjected to C18 (Thermo Fisher) desalting.

Sample preparation was performed in two phases due to biosafety issues. In the first phase, we processed samples from batches 1 to 8 including those collected at the first three or four time points. In the second phase, we processed samples from batches 9, 10, and 13–18, which included samples from the subsequent time points. In each phase, samples from three or four patients were randomly allocated to each batch. To monitor the reproducibility during the second round of sample preparation, 35 samples were analyzed as technical replicates in batches 13–15, including 29 samples from six COVID-19 patients, covering three to five time points. In addition, 10 samples from six COVID-19 patients at a randomly selected time point and eight control samples were randomly distributed in batches 9, 16–20. Pool-1 was the mixture of 120 samples in the first phase, while pool-2 was from 148 samples in the second phase. The protein ratios in batches 14–17 were thus further adjusted by the correction coefficient which is the ratio of pool-1 and pool-2.

TMT 16-plex (Thermo Fisher) reagents were used to label the digested peptides^39^. The TMT-labeled samples were further fractionated along a 2-h basic pH reverse phase liquid chromatography gradient using a Dionex Ultimate 3000 UHPLC (Thermo Fisher). Liquid chromatography–MS/MS analysis was performed using the Easy-nLC^TM^ 1200 system (Thermo Fisher) or a Dionex Ultimate 3000 RSLCnano system coupled to a Q Exactive HF or HF-X hybrid Quadrupole-Orbitrap (Thermo Fisher), along with a 60-min liquid chromatography gradient at a flowrate of 300 nL/min as previously described^10^. To reach comparable proteomics depth, the fractionated samples were combined into 30 fractions for analysis in QE-HF instruments and into 26 fractions for QE-HFX instruments.

### Database search and statistical analysis

MS data were analyzed using the Proteome Discoverer (version 2.4.1.15, Thermo Fisher)^40^ search engine against the human protein database downloaded from SwissProt (version 26/01/2020; 20375), with a precursor ion mass tolerance of 10 ppm, and fragment ion mass tolerance of 0.02 Da. Detailed parameters for the database searching can be found in a previous paper^10^. Briefly, TMT pro-plex labels at lysine residues and the N-terminus, and carbamidomethylation of cysteine residues were set as static modifications. A cut-off criterion of a *q*-value of 0.01, corresponding to a 1% FDR, was set for filtering-identified peptides with highly confident peptide hits.

After filtering proteins with 80% missing rate, 1252 proteins were used for differential expression analysis. The missing values were set to zero.

A two-sided unpaired Welch’s *t*-test was performed for each group comparison. The one-way analysis of variance (ANOVA) was used to determine the behavior of a variable in a dataset over eight or nine time points between the SC and LC groups. Adjusted *P*-values were calculated using the Benjamini and Hochberg correction.

### Metabolomic analysis

The pipeline for the metabolomics analysis, including sample preparation and quality control, was performed as previously described^10^. Metabolomics data were first normalized with the median of the intensity of some metabolites. Two-sided unpaired Welch’s *t*-test was used to compare each pair in the time series. Two-sided unpaired Welch *t*-test was performed to compare COVID-19 and non-COVID-19 patient groups.

### Mfuzz analysis

We applied ANOVA analysis to the proteomic and metabolomic data collected at nine time points (B-H adjusted *P*-value < 0.05) and selected 886 differentially expressed proteins and 314 differentially expressed metabolites. These proteins and metabolites were analyzed using Mfuzz (version 2.48.0) package^41^ in R (version 4.0.2) and classified into four groups, respectively.

### Pathway analysis

Four databases were used for the pathway enrichment analysis, including GO biological processes, KEGG pathway, Reactome, and canonical pathways. IPA (version 51963813) was then used to investigate the pathways corresponding to the differentially expressed proteins among the 1252 proteins we previously identified. The most significantly enriched pathways had a *P*-value < 0.01 and contained at least two proteins or metabolites from our dataset. We then used MetaAnalyst 5.0^42^ for the metabolomics pathway enrichment based on the 945 metabolites we previously identified.

### KNN network analysis

For great unbalanced number of molecules in the upregulated and downregulated groups, the screening approach of molecules that participated in the KNN network was different. This analysis was applied to the molecules that were differentially expressed between the SC and LC groups across nine (for the proteomics data) or eight (for the metabolomics data) time points tested by two-way ANOVA (Supplementary Table S4).

The distance matrices were calculated using the R function *dist* from the package *stats* (version 3.6.2). Each vertex contained a protein’s time series of intensities, and it was averaged on the samples. Each vertex *i* connected to *k*-nearest neighbors, and the distances between them were calculated by Euclidean distance. For a directed KNN network, all the vertices had the same out-degree (*k*) but a variable in-degree. An undirected network made *A*_*j,i*_ = 1 when *A*_*i,j*_ = 1 in the adjacency matrix. Small *k* (for instance, *k* = 5) demonstrated relatively small groups of proteins with similar time series trend. The undirected networks were plotted with *igraph* (version 1.2.5), where the width of the line represented the distance between two proteins via the Fruchterman–Reingold method.

### Random forest analysis

The features were selected from 1229 molecules including 808 proteins and 421 metabolites with standard deviation < 1 in the training dataset. Then the data matrix was normalized using *Z*-score. We firstly selected 420 molecules including 323 proteins and 97 metabolites using random forest. Then 22 molecules were screened after six-fold cross validation. Thus, we built a 10-molecule classifier including nine proteins and one metabolite to distinguish LC and SC groups. We then validated the classifier in the independent test dataset^10^. The machine learning was performed using the R package randomForest (version 4.6.14) as described previously with some modifications as described^10^. We optimized the key random forest parameters including the cutoff values for decrease mean accuracy, cross-validation fold, and the number of trees. Input protein features were selected based on the mean decrease accuracy cutoff. For the optimized model, the minimal mean decrease accuracy of protein features was set as 1 for the 420-feature selection and 3 for the 22-feature selection, the mtry was set as 3, and 1000 trees were built.

### Flow cytometry analysis

Direct immunofluorescence was used for immune cell detection, while the indirect method was used for cytokines quantification, following the manufacturer’s instructions. In brief, 50 μL peripheral blood samples with EDTA anticoagulants (within 4 h after collection) were incubated with mixed antibodies including CD4-PE-Cy7 (UB105441, UB Biotechnology Co., Ltd., Hangzhou, China), CD3-FITC (UB104411), CD25-PE (UB112421), CD45-PerCP-Cy5.5 (UB109481), and CD127-APC (UB113451), for 15 min at room temperature in darkness. 450 μL hemolysin was used to destroy erythrocytes. The labeled immune cells were then counted by flow cytometry.

After immune cells labeling, the same blood samples were centrifuged at 1000× *g* for 10 min. The isolated plasma samples were used for the detection of cytokines using a kit (UB08PX), including IL-2, IL-4, IL-5, IL-6, IL-10, IL-17A, TNF-α, and IFN-γ. The plasma samples were incubated with microspheres coated with anti-cytokine specific primary antibodies for 2 h, mixed with anti-cytokine specific secondary antibodies labeled with biotin for 1 h, and then with 25 μL streptomavidin-phycoerythrin (SA-PE) for 30 min at room temperature in darkness. The resuspended cytokines could be assayed by flow cytometry after removing the supernatant by centrifugation at 250× *g* for 5 min.

Double negative and single-stain controls were prepared from normal samples and used to calculate a compensation matrix. Sample acquisition was performed on a Gallios cytometer (Beckman Coulter). Final analysis and graphical output were performed using NovoExpress software (Agilent Bio).

## Supplementary information

Supplementary information

## Data Availability

All data are available in the manuscript or the supplementary information. The proteomics data are deposited in ProteomeXchange Consortium (https://www.iprox.org/). Project ID is IPX0002170000. The link to access the raw data is https://www.iprox.cn/page/project.html?id=IPX0002170000. All the codes used in this study are provided in Github with a link https://github.com/guomics-lab/CVDTSA.
