# Supplementary material for: Enhanced inflammation and suppressed adaptive immunity in COVID-19 with prolonged RNA shedding
Source: Cell Discov. 2022 Jul 25;8:70. doi: 10.1038/s41421-022-00441-y (PMC9311354; doi:10.1038/s41421-022-00441-y)
Supplement: Supplementary file 1 — Supplementary information [file 41421_2022_441_MOESM1_ESM.pdf]

Supplementary Information

Supplementary Fig. S1

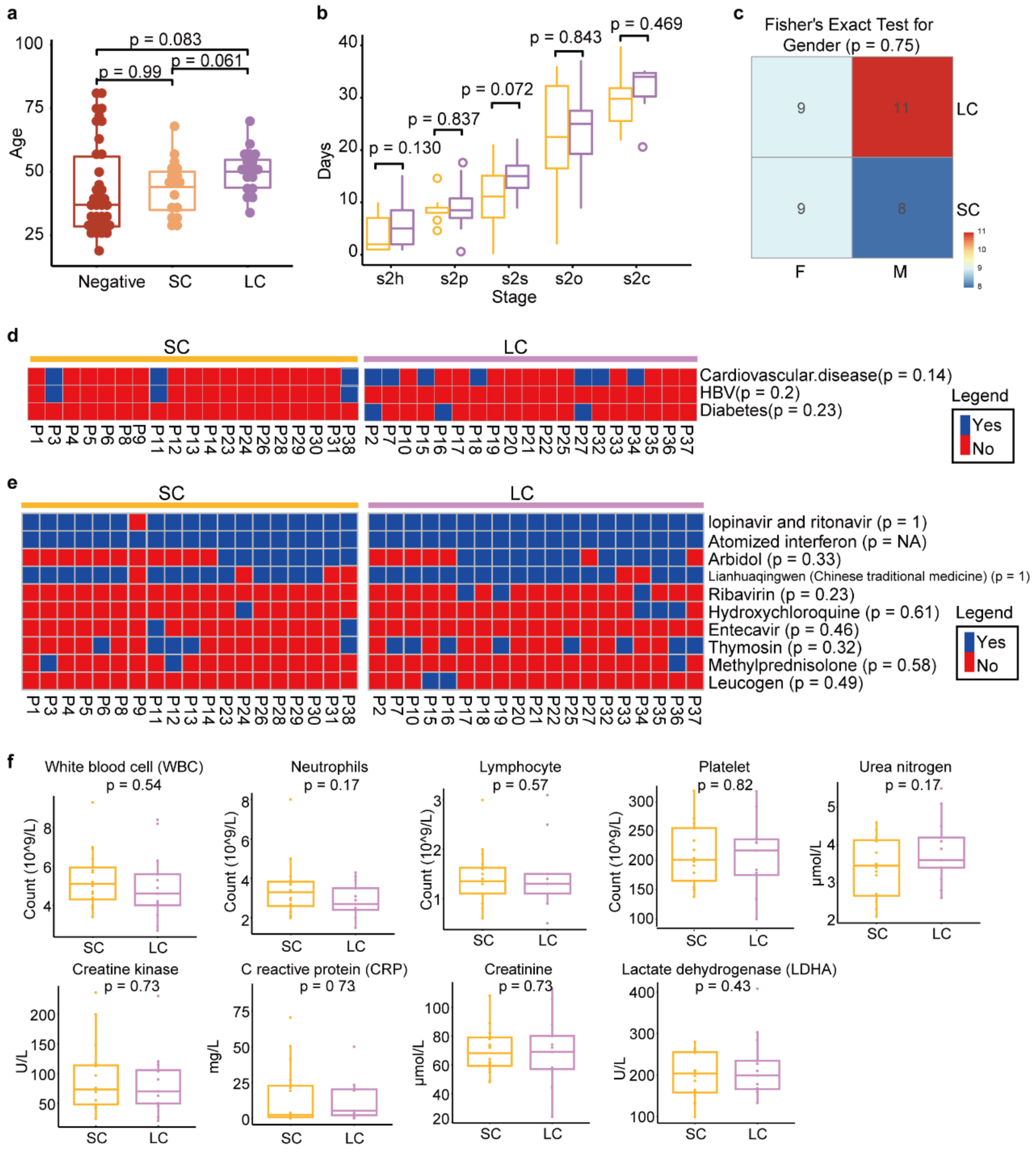

Supplementary Fig. S1 Correlation analysis between the SC and LC groups based on clinical data.

(a) Boxplot representing the ages of the controls (N=35), and the LC (N=19) and SC (N=19) groups analyzed by Wilcoxon rank-sum test. **(b)** Boxplot of the durations of five disease stages analyzed by Wilcoxon rank-sum test: S2h,

onset to hospitalization; s2p, onset to peak time of pneumonia; s2s, onset to start of absorption of pneumonia; s2o, onset to obvious pneumonia absorption; s2c, onset to complete pneumonia absorption. **(c)** Fisher’s exact test for gender between the SC and LC groups. **(d)** Heatmaps showing the comorbidities of the SC and LC groups. The column set on the right lists the comorbidities and their corresponding p-values, indicating the comorbidities differences between the two groups (Fisher’s exact test). **(e)** Heatmaps showing the medication history of the SC and LC groups. The column set on the right lists the drugs and their corresponding p-values, indicating the medication difference between the two groups (Fisher’s exact test). **(f)** Blood test results for the SC (N=17) and LC (N=15) patients. The boxplots display the blood biochemistry tests based on samples collected on the day of hospitalization (Welch’s t-test).

Supplementary Fig. S2

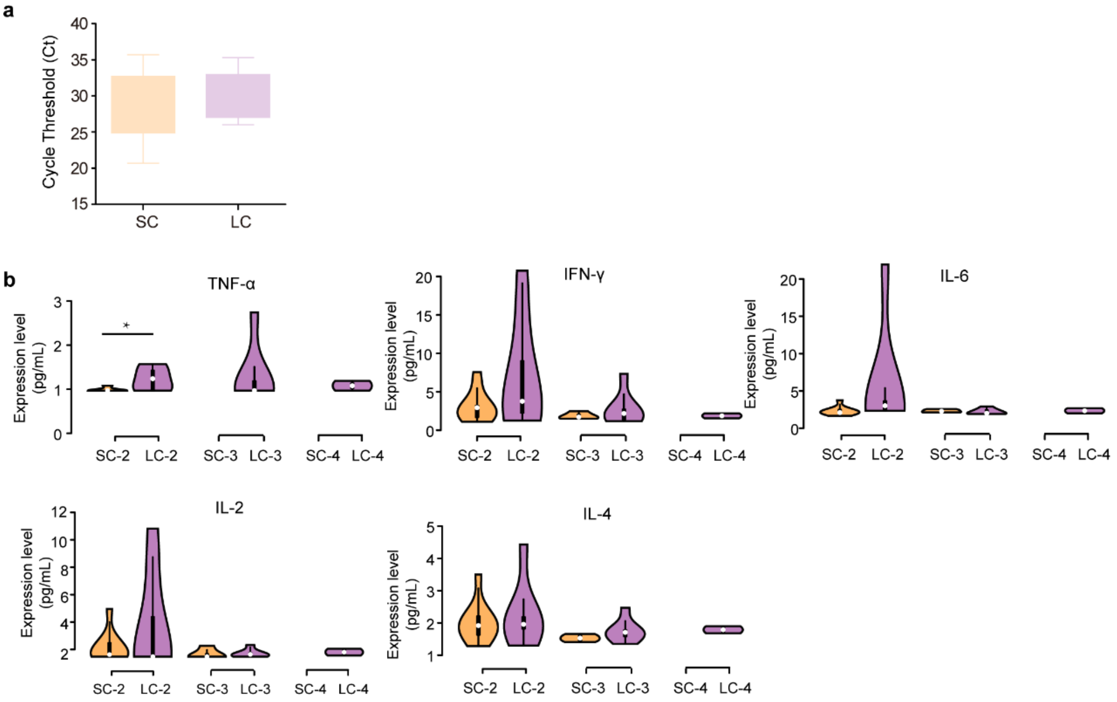

Supplementary Fig. S2 Measurement of virus load, cytokines, and antibodies, in the SC and LC groups.

(a) Boxplot of the relative quantification of SARS-CoV-2 virus RNA detected by RT-PCR (p-value from independent sample t-test). **(b)** Violin plots of the expression levels of cytokines detected by antibody-based flow cytometry analysis (Week 2: 9 SC samples, 6 LC samples; week 3: 11 SC samples, 8 LC samples; week 4: 4 SC samples, 5 LC samples; total: 24 samples from 13 SC patients and 19 samples from 12 LC patients). The y-axis shows the intensity of each cytokine (p-value from independent sample t-test).

Supplementary Fig. S3

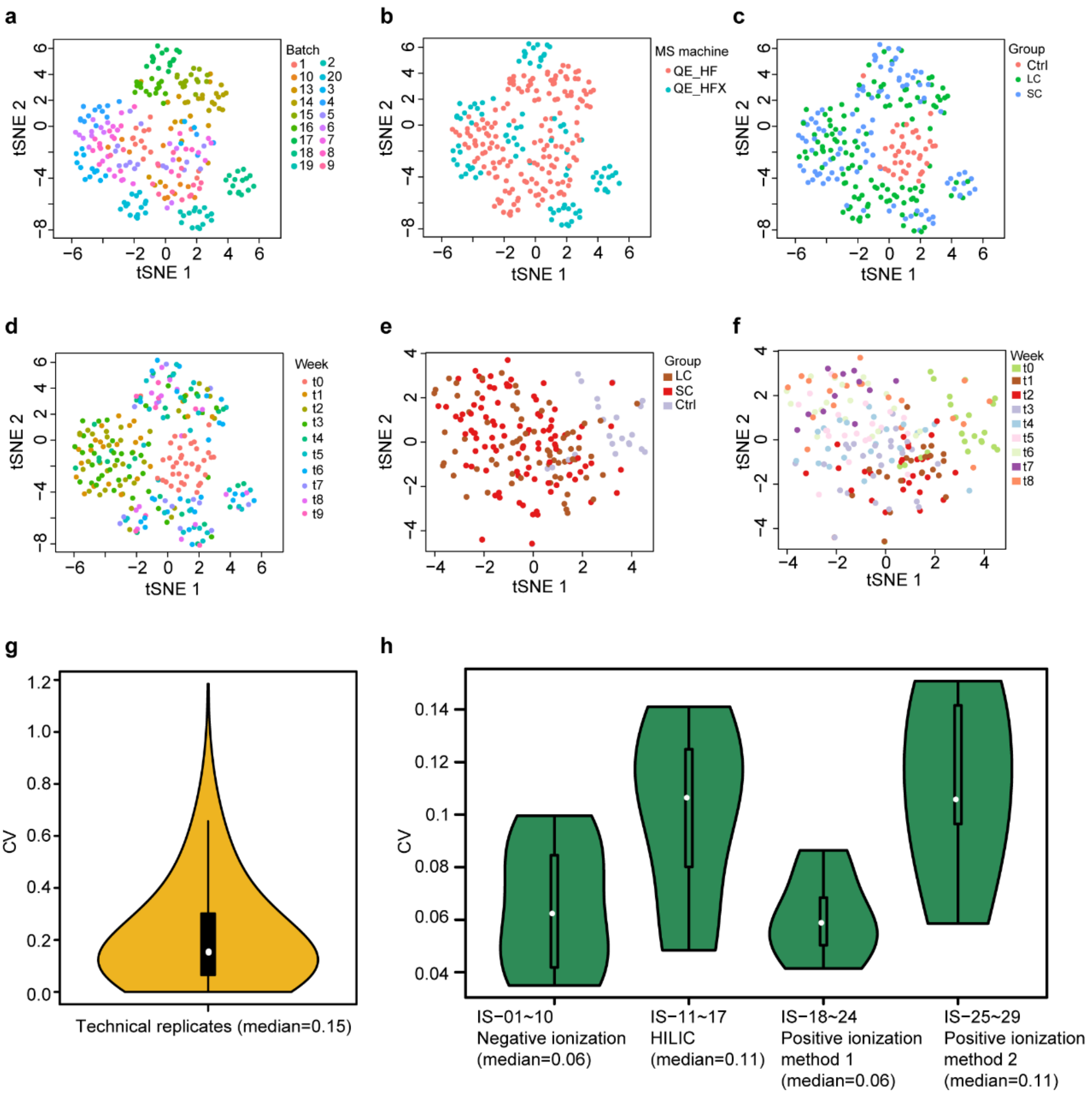

Supplementary Fig. S3 Quality assessment of the proteomics data.

(a)-(d) tSNE plots of the proteomics data using BatchServer[1], color-coded based on the batch (A), the mass spectrometer used (B), patient groups (C), and the acquisition time (D). **(e)-(f)** tSNE plots performed for the metabolomics data, color-coded based on the patient group (E), and the acquisition time (F). **(g)** The median CV of the proteomics data is calculated by the abundance of the quantified proteins in the 44 technical replicates and the 18 pool samples. **(h)** The median CV of the metabolomics data is

calculated by the abundance of quantified proteins in 29 technical replicates acquired by four different methods: negative ionization, HILIC column, positive ionization with the mobile phase including methanol and water (group 1), and another positive ionization with the mobile phase including methanol, acetonitrile, and water (group 2).

Supplementary Fig. S4

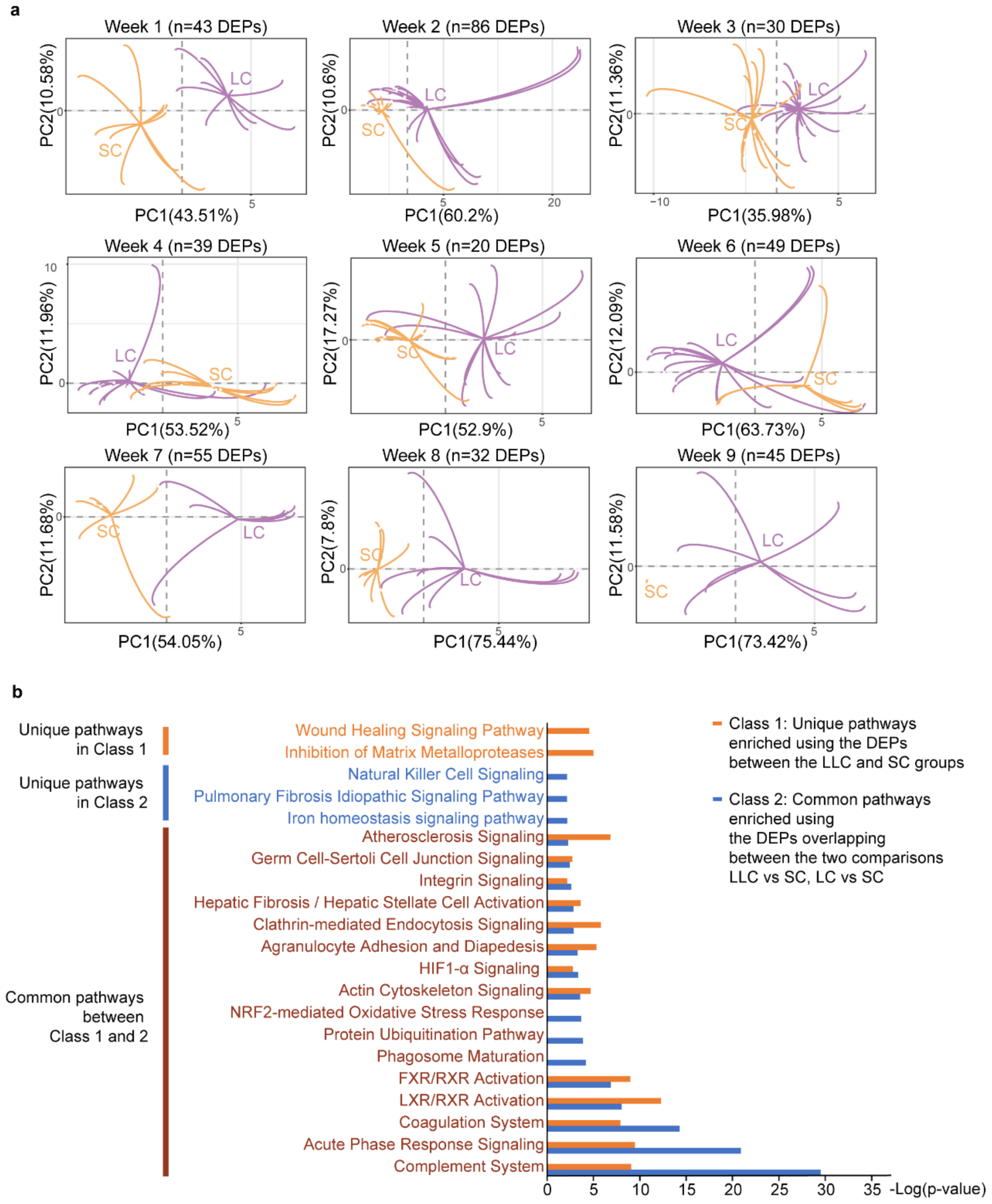

Supplementary Fig. S4 Differentially expressed analysis between the SC, LC and LLC groups based on proteomic data.

(a) These plots present the distances of the host responses of the LC and SC samples in principal component analysis (PCA). The position of centroid is the average value of the PC1 and PC2 coordinates of each sample in a patient

group. The line connecting the centroid and each sample represents their distance. **(b)** Two classes pathway enrichment were performed. The class 1 was using 268 commonly differentially expressed proteins (DEPs) between the two comparisons, LLC vs SC and LC vs SC. The class 2 was using 115 uniquely DEPs between the LLC and SC. Blue columns represent class 1 pathway enrichment, while red columns represent class 2. The names of pathways labeled with brown, blue and red were common pathways between the class 1 and 2, unique pathways in the class 2 and unique pathways in the class 1, respectively.

68 **Supplementary Fig. S5**

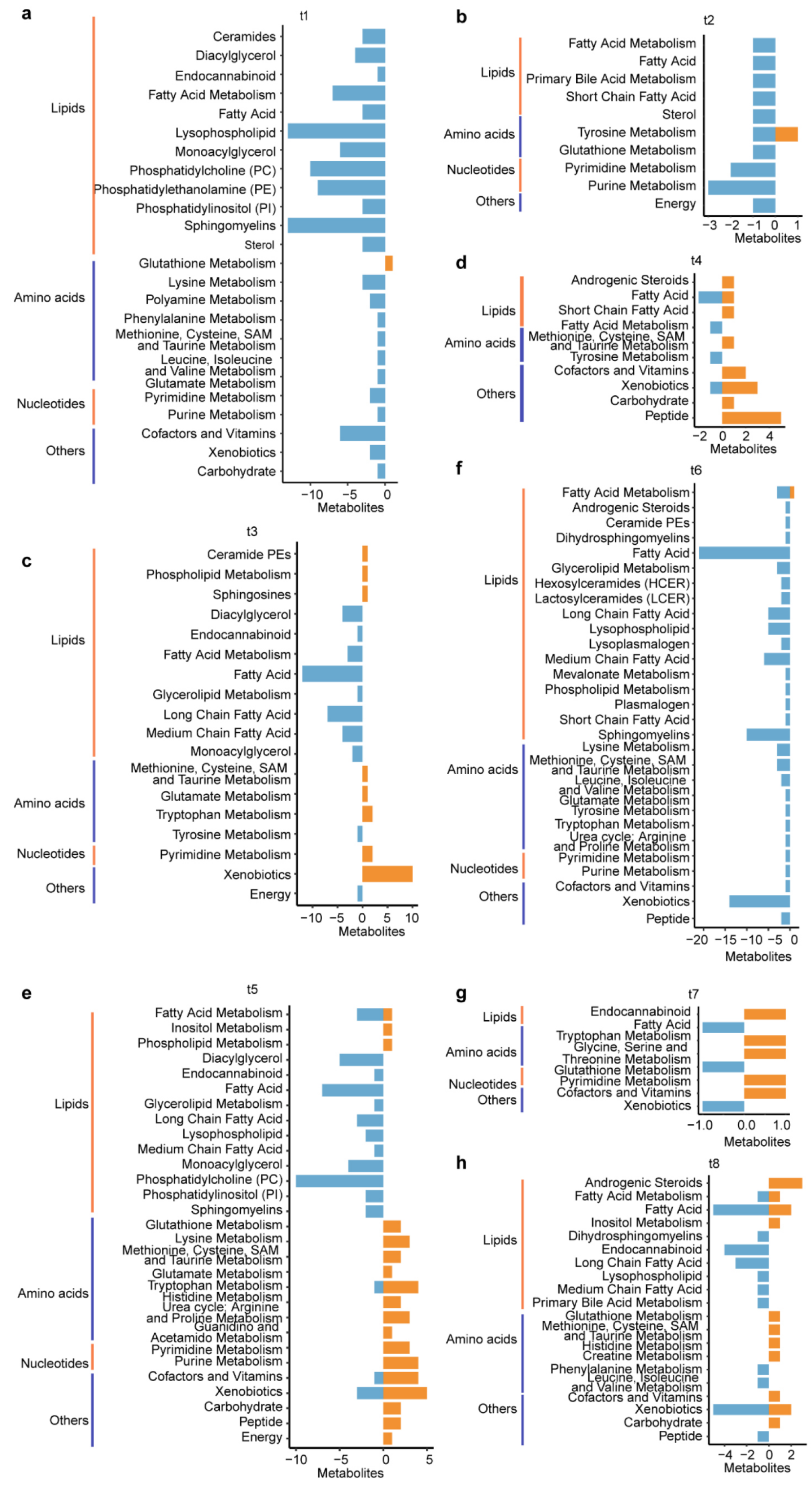

69

Supplementary Fig. S5 Different dynamic metabolomics characteristics between the SC and LC groups.

(a)-(h) Stacked bar charts showing the distribution of dysregulated expression metabolites sub-classifications between the LC and SC groups (Orange: upregulated metabolites in the LC group, blue: downregulated metabolites in the LC group, $|\log2(FC)| > 0.25$, Welch’s t-test $p < 0.05$).

Supplementary Fig. S6

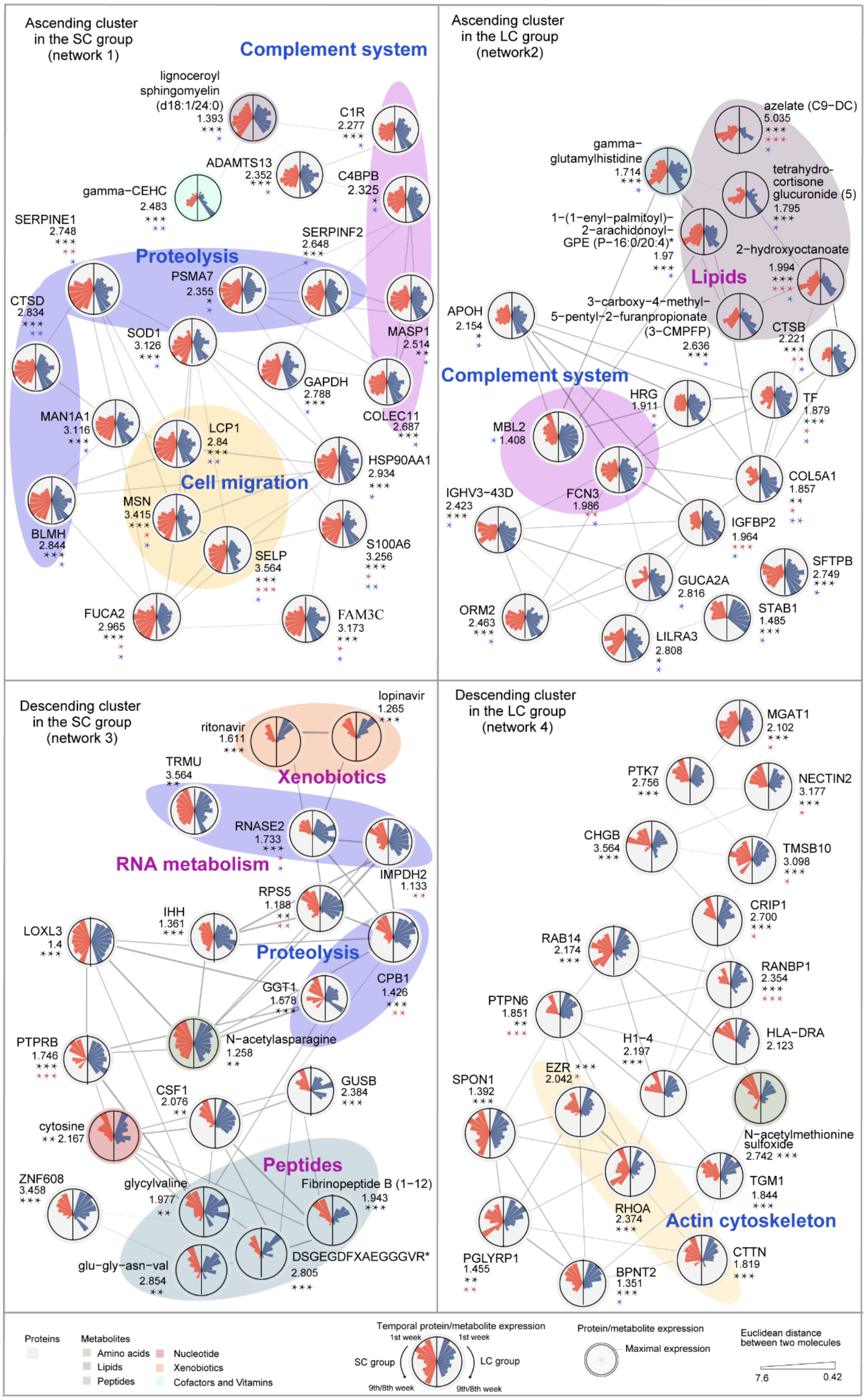

Supplementary Fig. S6 Complex interaction networks of proteome and metabolome.

The correlation relationship of the proteins and metabolites are shown for the SC and LC groups. The top left corner panel includes the persistently ascending molecules from the SC group. The top right corner panel includes the persistently ascending molecules from the LC group. The lower left panel includes the persistently descending molecules from the SC patient group. The lower right panel includes the persistently descending molecules from the LC group. The right/left half of each circle shows the expression time-series in the LC/SC group (two-way ANOVA; p-value: * <0.05; ** <0.01; *** <0.001). Black asterisks indicate a significant variance over 8-9 time points in the SC or LC groups; the red asterisks represent the difference between the LC and SC groups; the blue asterisks represent the interaction difference between time points and the LC and SC groups. The outermost ring represents the maximum abundance of the proteins/metabolites, between the SC and LC groups, across nine time points for proteins and eight time points for metabolites. The different backgrounds represent the classifications of the molecules. The top pathways were enriched using all molecules in the four clusters, using MetaboAnalyst (p-value < 0.05).

Supplementary Fig. S7

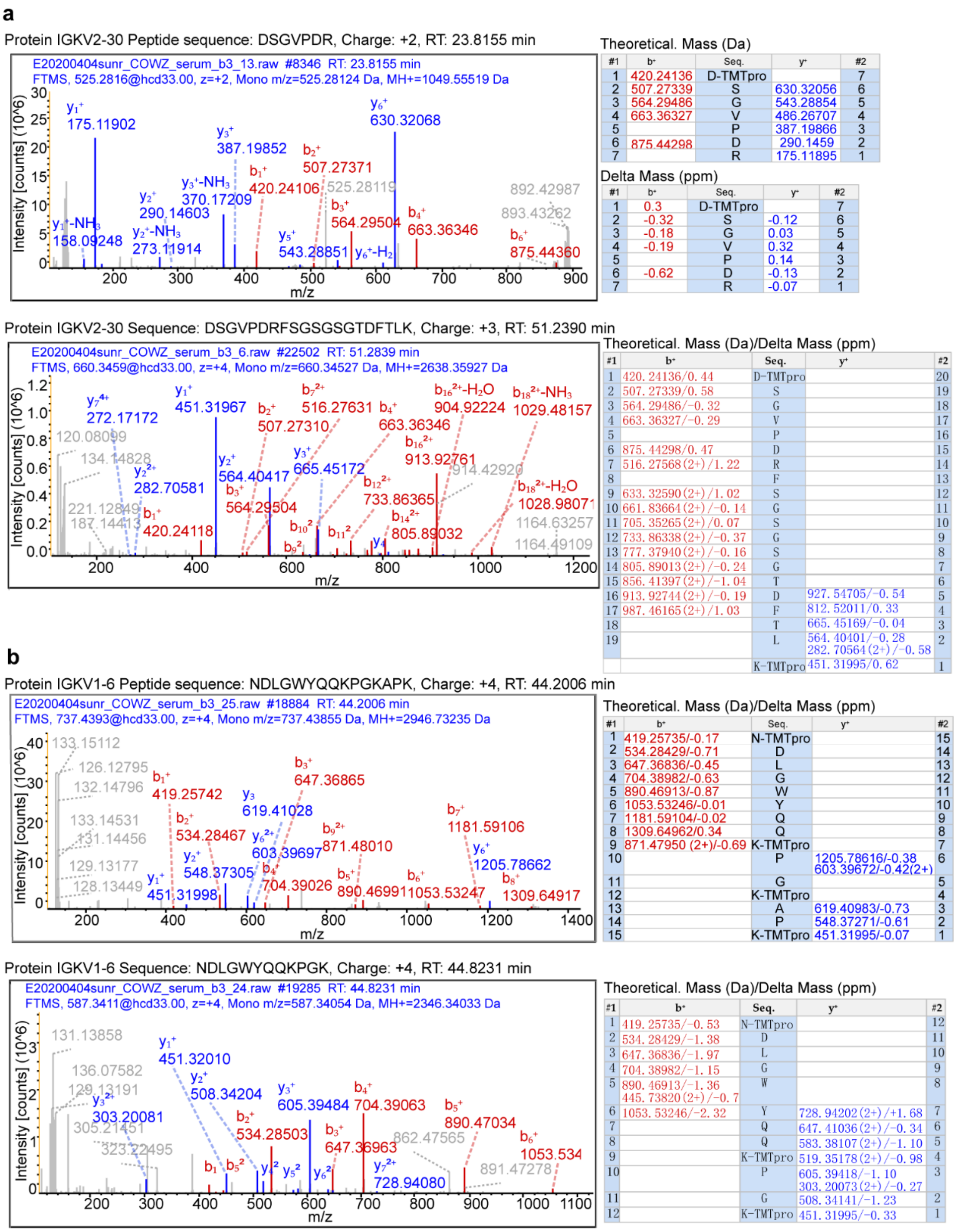

Supplementary Fig. S7 The MS/MS spectra of the unique peptides of IGKV2-30 (a) and IGKV1-6 (b).

Reference

1 Zhu, T. *et al.* BatchServer: A Web Server for Batch Effect Evaluation, Visualization, and Correction. *J Proteome Res* **20**, 1079-1086, doi:10.1021/acs.jproteome.0c00488 (2021).
